# Virulence Factors and Antibiotic Susceptibility of *Staphylococcus aureus* Isolates in Ready-to-Eat Foods: Detection of *S. aureus* Contamination and a High Prevalence of Virulence Genes

**DOI:** 10.3390/ijerph13020199

**Published:** 2016-02-05

**Authors:** Suat Moi Puah, Kek Heng Chua, Jin Ai Mary Anne Tan

**Affiliations:** Department of Biomedical Science, Faculty of Medicine, University of Malaya, Kuala Lumpur 50603, Malaysia; suatmoi@um.edu.my (S.M.P.), khchua@um.edu.my (K.H.C.)

**Keywords:** antibiotic resistance, sashimi, *Staphylococcus aureus*, sushi, virulence genes

## Abstract

*Staphylococcus aureus* is one of the leading causes of food poisoning. Its pathogenicity results from the possession of virulence genes that produce different toxins which result in self-limiting to severe illness often requiring hospitalization. In this study of 200 sushi and sashimi samples, *S. aureus* contamination was confirmed in 26% of the food samples. The *S. aureus* isolates were further characterized for virulence genes and antibiotic susceptibility. A high incidence of virulence genes was identified in 96.2% of the isolates and 20 different virulence gene profiles were confirmed. DNA amplification showed that 30.8% (16/52) of the *S. aureus* carried at least one SE gene which causes staphylococcal food poisoning. The most common enterotoxin gene was *seg* (11.5%) and the *egc* cluster was detected in 5.8% of the isolates. A combination of *hla* and *hld* was the most prevalent coexistence virulence genes and accounted for 59.6% of all isolates. Antibiotic resistance studies showed tetracycline resistance to be the most common at 28.8% while multi-drug resistance was found to be low at 3.8%. In conclusion, the high rate of *S. aureus* in the sampled sushi and sashimi indicates the need for food safety guidelines.

## 1. Introduction

The term ready-to-eat (RTE) foods refers to prepared food products that are sold as perishable meals for immediate consumption, refrigerated or frozen foods that require minimal cooking, or shelf-stable products [1]. The popularity of the different types of RTE foods differs from country to country, depending on the staple diets of the population. In Malaysia, RTE foods like delicatessen meat and dairy products are unpopular because of their high cost and mainly because rice is the standard diet of Malaysians. Thus, Japanese RTE foods such as sushi (vinegar seasoned rice combined with a variety of toppings) and sashimi (raw meat or fish slivers) are more readily accepted as quick, affordable and tasty meals that require no further cooking.

Although sushi and sashimi are convenient nutritious meals, questions have been raised with regards to their safety and microbiological quality as these RTE foods do not undergo any additional treatment or cooking steps to ensure safety prior to consumption. Food safety issues associated with the consumption of sushi and/or sashimi contaminated with *Staphylococcus aureus* have been reported in U.S.A. [2], Hong Kong [3], Germany [4], Japan [5] and Italy [6]. Staphylococcal food intoxication results from the ingestion of pre-formed enterotoxins produced by *S. aureus* [7]. Symptoms show a rapid onset with nausea, vomiting and abdominal cramping, with or without diarrhea [8]. The foodborne illness is usually self-limiting and resolves within 24 to 48 h after onset. However, occasionally the diseases can be severe and hospitalization is needed. The actual incidence of illness may be higher than reported mainly due to misdiagnosis or under-reported cases [9].

*S. aureus* possesses many virulence factors and the most notable are the five major classical types of staphylococcal enterotoxins (SEs: SEA to SEE), the non-classical SE-like toxins (SEl: SEG to SEU), and other virulence genes such as toxic shock syndrome toxin 1 (TSST-1), exfoliative toxins and cytolytic toxins (leukocidin and hemolysins). Staphylococcal enterotoxins (SEs) are heat stable proteins that are mainly associated with food poisoning outbreaks [7,10], while TSST-1 is a superantigenic exotoxin that causes toxic shock syndrome [11]. The exfoliative toxins are responsible for staphylococcal scalded skin syndrome that typically affects infants and young children [12], lukPV cytotoxin causes leukocytosis with necrotic lesions in the skin or mucosa [13] while hemolysins involve epithelial barrier disruption [14].

Hence, the objectives of the present study were to isolate *S. aureus* in sushi and sashimi sampled from different food outlets located in the Klang Valley in Malaysia, and subsequently to characterize the presence of virulence gene(s) and antibiotic resistance patterns in these *S. aureus* isolates.

## 2. Materials and Methods

### 2.1. Sample Collection and Isolation of S. aureus

The Klang Valley, with its population of over 7 million inhabitants, was selected for this study as it comprises the capital city, Kuala Lumpur, and neighboring suburbs with varied businesses, from commercial hypermarkets and shopping malls to smaller chain retail outlets and restaurants. Retail RTE sushi (*n* = 149) and sashimi (*n* = 51) were collected from different food outlets in the Klang Valley, Malaysia between August and December 2014. Various types of sushi with different toppings—marine fishes, fish roe, squid, octopus, jellyfish, edible seaweed, scallop, egg, crab stick, cherry shrimp, prawn and clam were selected for this study. Sashimi samples consisted of salmon, tuna, yellow tail, squid and scallop cut into slivers.

The exterior surface of the RTE package was cleaned with 70% (*v*/*v*) alcohol before sample isolation. Each sample was aseptically weighed in an analytical balance and twenty-five grams was placed into a sterile plastic bag. The plastic bag then received with 225 mL of buffered peptone water (Oxoid, Hampshire, UK) and homogenized in a Stomacher Bagmixer 400W (Interscience, Saint-Nom, France) for one minute. The mixture was then incubated for 16 h at 37 °C with shaking at 100 rpm. Five milliliter aliquot of the enriched homogenate was transferred into 50 mL trypticase soy broth (Oxoid) containing 7.5% NaCl. After incubation at 35 °C for 18 h, a loopful of the culture was plated onto Baird-Parker agar supplemented with egg yolk tellurite emulsion (Oxoid) and incubated overnight at 37 °C. Three presumptive *S. aureus* colonies (black shiny colonies surrounded by a clear halo) per food sample were randomly selected for purification using trypticase soy agar (TSA) (Oxoid) containing 0.6% yeast extract.

### 2.2. DNA Extraction and Identification of S. aureus

Total genomic DNA was prepared using an adapted in-house boiling method [15] and stored at −20 °C for further investigations. All presumptive colonies were confirmed by DNA amplification using the polymerase chain reaction (PCR) for *S. aureus* with two sets of primers targeting the 16S rRNA (*Staphylococcus* genus-specific, 228 bp) [16] and *nuc* (*S. aureus* species-specific, 279 bp) genes [17]. The primer sequences used for DNA amplification are—16S rRNA (F): 5′-GTAGGTGGCAAGCGTTATCC-3′, 16S rRNA (R): 5′-CGCACATCAGCGTCAG-3′; *nuc* (F): 5′-GCGATTGATGGTGATACGGTT-3′ and *nuc* (R): 5′-AGCCAAGCCTTGACGA-ACTAAAGC- 3′.

### 2.3. DNA Amplification of S. aureus Virulence Genes

The presence of 32 virulence genes—20 enterotoxin genes (*sea, seb, sec, sed, see, seg, seh, sei, sej*, *sek,* *sel, sem, sen, seo, sep, seq, ser, ses, set, seu*)*,* toxic shock syndrome gene (*tst*)*,* three exofoliative toxin genes (*eta, etb, etd*), three leukocidin encoding genes (*lukM, lukED, lukPV*) and five hemolysin encoding genes (*hla, hlb, hld, hlg, hlgv*)—were investigated by singleplex PCR assay [13,18,19,20,21,22,23,24,25,26]. DNA amplifications were carried out using primers as described in published studies (Supplementary Table S1). Each singleplex PCR was performed using TopTaq Master Mix Kit (Qiagen, Hilden, Germany) according to the manufacturer’s protocol. DNA amplification was carried out in a PCR mixture that contained 25 μL TopTaq Master Mix, 0.2 μM forward primer, 0.2 μM reverse primer, 100 ng of DNA template, RNase-free water PCR products were then electrophoresed in a 1.5% (*w*/*v*) agarose gel. One representative of each positive virulence gene isolate was confirmed by direct DNA sequencing.

### 2.4. Antimicrobial Susceptibility Testing

The susceptibility of the *S. aureus* isolates to antimicrobials was tested by the disc diffusion method on Mueller-Hinton agar using commercial antibiotic disks (Oxoid) according to Clinical and Laboratory Standards Institute (CLSI) guidelines [27]. *S. aureus* American Type Culture Collection (ATCC) 29213 and *Escherichia coli* ATCC 25922 were used as quality control strains in each run. Ten antimicrobials were tested: cefoxitin (30 µg), gentamicin (10 µg), amikacin (30 µg), tetracycline (30 µg), vancomycin (30 µg), erythromycin (15 µg), ciprofloxacin (5 µg), trimethoprim/sulfamethoxazole (1.25/23.75 µg), chloramphenicol (30 µg) and cefoperazone (75 µg). Interpretation of inhibition zones was carried out based on the manufacturers’ and CLSI guidelines [27].

## 3. Results

### 3.1. Isolation and Identification of Isolates

Of the 200 food items, the samples consisted of 74.5% (149/200) sushi and 25.5% (51/200) sashimi. *S. aureus* was detected in 26% (52/200) of the collected retail food items. The contaminated food items consisted of 32 sushi and 20 sashimi samples.

### 3.2. Virulence Potential

**Figure 1:**
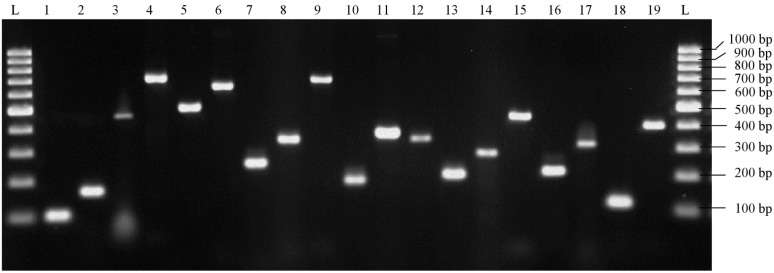
Gel electrophoresis of amplified virulence gene products from food isolates. Lane L: 100 bp molecular weight marker; lane 1: *sea* (102 bp); lane 2: *seb* (164 bp); lanes 3: *sec* (451 bp); lane 4: *seg* (704 bp); lane 5: *seh* (495 bp); lane 6: *sei* (630 bp); lane 7: *sel* (240 bp); lane 8: *sem* (326 bp); lane 9: *sen* (680 bp); lane 10: *seo* (180 bp); lane 11: *ser* (367 bp); lane 12: *tsst* (326 bp); lane 13: *eta* (190 bp); lane 14: *lukDE* (269 bp); lane 15: *lukPV* (433 bp); lane 16: *hla* (209 bp); lane 17: *hlb* (309 bp); lane 18: *hld* (111 bp); lane 19: *hlgv* (390 bp).

Of the 52 *S. aureus* isolates, 50 (96.2%) were positive for one or more virulence genes (Table 1) and 20 different virulence gene profiles were confirmed (Table 2). None of the 52 isolates possessed any of the *sed, see, sej, sek, sep, seq, ses, set, seu, etb, etd, lukM* and *hlg* virulence genes. The amplified fragments of the 19 virulence genes corresponded to the expected published amplicon sizes as shown in Supplementary Table S1 and Figure 1.

**Table 1:** Prevalence of virulence genes in 52 *Staphylococcus aureus* isolated from sushi and sashimi samples.

Virulence Gene Profiles		Sushi *n* = 32 *n* (%)	Sashimi *n* = 20 *n* (%)	Total *n* = 52 *n* (%)
One or more		31 (96.9)	19 (95.0)	50 (96.2)
Classical	*sea*	0	3 (15.0)	3 (5.8)
SE genes	*seb*	1 (3.1)	0	1 (1.9)
	*sec*	3 (9.4)	0	3 (5.8)
Non-classical	*seg*	6 (18.8)	0	6 (11.5)
SE genes	*seh*	1 (3.1)	0	1 (1.9)
	*sei*	4 (12.5)	0	4 (7.7)
	*sel*	2 (6.3)	0	2 (3.8)
	*sem*	4 (12.5)	0	4 (7.7)
	*sen*	3 (9.4)	0	3 (5.8)
	*seo*	4 (12.5)	0	4 (7.7)
	*ser*	0	4 (20.0)	4 (7.7)
Others	*tsst*	4 (12.5)	1 (5.0)	5 (9.6)
	*eta*	2 (6.3)	0	2 (3.8)
	*lukDE*	15 (46.9)	5 (25.0)	20 (38.5)
	*lukPV*	4 (12.5)	1 (5.0)	5 (9.6)
	*hla*	28 (87.5)	19 (95.0)	47 (90.4)
	*hlb*	0	5 (25.0)	5 (9.6)
	*hld*	31 (96.9)	0	31 (59.6)
	*hlgv*	11 (34.4)	5 (25.0)	16 (30.8)
No gene		1 (3.1)	1 (5.0)	2 (3.8)

**Table 2:** Virulence gene profiles in 52 *Staphylococcus aureus* isolated from sushi and sashimi samples.

Virulence Gene Profiles	Sushi *n* = 32 *n* (%)	Sashimi *n* = 20 *n* (%)	Total *n* = 52 *n* (%)
*hla*	0	9 (45.0)	9 (17.3)
*hld*	1 (3.1)	0	1 (1.9)
*pvl, hla*	0	1 (5.0)	1 (1.9)
*ser, hla*	0	3 (15.0)	3 (5.8)
*hla, hld* *	8 (25.0)	0	8 (15.4)
*hld, lukDE*	2 (6.3)	0	2 (3.8)
*eta, hla, hld* *	1 (3.1)	0	1 (1.9)
*hla, hld, lukDE* *	2 (6.3)	0	2 (3.8)
*pvl, hla, hld* *	1 (3.1)	0	1 (1.9)
*seg, hla, hld* *	2 (6.3)	0	2 (3.8)
*tsst, ser, hla*	0	1 (5.0)	1 (1.9)
*hla, hld, hlgv, lukDE* *	5 (15.6)	2 (10.0)	7 (13.5)
*pvl, hla, hld, hlgv, lukDE* *	2 (6.3)	0	2 (3.8)
*sea, hla, hld, hlgv, lukDE* *	1 (3.1)	2 (10.0)	3 (5.8)
*seb, seh, pvl, hla, hld* *	1 (3.1)	0	1 (1.9)
*sec, hla, hld, hlgv, lukDE* *	2 (6.3)	0	2 (3.8)
*seg, sei, sen, seo, sem, tsst, hla, hld* *	1 (3.1)	0	1 (1.9)
*seg, sei, sen, seo, sem, tsst, eta, hla, hlgv*	1 (3.1)	0	1 (1.9)
*seg, sei, seo, sem, tsst, sel, hla, hlgv, lukDE*	1 (3.1)	0	1 (1.9)
*sec, seg, sei, sen, seo, sem, tsst, sel, hla, hld, hlgv, lukDE* *	1 (3.1)	0	1 (1.9)

* *hla-hld* combination.

Notably, the non-classical SE gene (*seg*) was most frequently detected at 11.5% followed by *sei*, *sem*, *seo* and *ser* (7.7% each) (Table 1). The *egc* cluster (*seg-sei-sem-sen-seo*) was observed in 3 isolates (5.8%) with other additional genes (Table 2). Isolates carrying non-SE/SEl genes were observed in the following order: *hla* (90.4%), *hld* (59.6%), *lukDE* (38.5%), *hlgv* (30.8%), *tsst* (9.6%), *lukPV* (9.6%), *hlb* (9.6%) and *eta* (3.8%). Thirty-one isolates (59.6%) harboured the most common combination of virulence genes—*hla* and *hld* either together or in combination with other genes.

### 3.3. Susceptibility of S. aureus to Antimicrobials

Overall, 34.6% (18/52) of the *S. aureus* isolates exhibited resistance phenotypes to at least one antibiotic (Table 3). Resistance to tetracycline was most commonly observed (28.8%). A small percentage of between 1.9% and 7.7% of the isolates demonstrated resistance to gentamicin, amikacin, cefoperazone, trimethoprim-sulfamethoxazole, chloramphenicol, vancomycin, ciprofloxacin cefoxitin and erythromycin.

**Table 3:** Frequency of antimicrobial resistance in 52 *Staphylococcus aureus* isolates recovered from sushi and sashimi.

Antimicrobial Agents	Disk Concentration (µg)	Number of Resistant Isolates (%)		
		Sushi (*n* = 32)	Sashimi (*n* = 20)	Total (*n* = 52)
Cefoxitin (FOX)	30	3 (9.4)	1 (5.0)	4 (7.7)
Gentamicin (CN)	10	0	1 (5.0)	1 (1.9)
Amikacin (AK)	30	0	1 (5.0)	1 (1.9)
Tetracycline (TE)	30	3 (9.4)	12 (60.0)	15 (28.8)
Vancomycin (VA)	30	2 (6.3)	1 (5.0)	3 (5.8)
Erythromycin (E)	15	2 (6.3)	2 (10.0)	4 (7.7)
Ciprofloxacin (CIP)	5	1 (3.1)	2 (10.0)	3 (5.8)
Trimethoprim-sulfamethoxazole (SXT)	1.25/23.7	1 (3.1)	1 (5.0)	2 (3.8)
Chloramphenicol (C)	30	1 (3.1)	1 (5.0)	2 (3.8)
Cefoperazone (CFP)	75	0	1 (5.0)	1 (1.9)
One antibiotic		5 (15.6)	13 (65.0)	18 (34.6)
Two antibiotics		1 (3.1)	0	1 (1.9)
Six antibiotics		1 (3.1)	0	1 (1.9)
Ten antibiotics		0	1 (5.0)	1 (1.9)

## 4. Discussion

This study showed that the overall prevalence of *S. aureus* in the 200 sushi and sashimi samples was high at 26%. *S. aureus* occurrence from sushi and sashimi samples showed a high rate at 21.5% (32/149) and 39.2% (20/51) respectively; this can be a result from contamination through human contact as *S. aureus* is not part of the normal microflora of freshly caught marine fishes and fishes bred in farms [28]. Of the 32 contaminated sushi samples, 22 samples were topped with raw seafood products, *i.e.*, salmon (*n* = 13), fish roe (*n* = 5), tuna (*n* = 3) and squid (*n* = 1). In a report by Vazquez-Sanchez *et al.* [29], *S. aureus* was also reported to be responsible for a significant proportion (25.2%) of contamination in fishery products marketed in Northwest Spain. *S. aureus* is recognized as common resident skin and nasal microbiota in humans, thus these are possible sources of contamination [8]. As the preparation of sushi and sashimi involves a great deal of handling with bare or gloved hands, a possible way to determine definite sources of contamination will be to collect hand and nasal swabs from food handlers.

Investigations carried out to assess the quality and safety of sushi and/or sashimi showed *S. aureus* contamination in sushi (15.79%) and sashimi (32.79%) samples collected from restaurants in Italy and northern Portugal respectively [6,30]. Recently, Hammad and co-workers [5] demonstrated a high prevalence (87%) of *S. aureus* contamination in 200 sashimi samples collected in Hiroshima (Japan). The results from this study thus concur with previous published reports.

In Malaysia, there is currently no documented outbreak of foodborne disease due to sushi consumption. The 52 *S. aureus* isolated in this study were also screened for virulence genes to gain insight into their pathogenic potential. Overall, nearly all of the *S. aureus* isolates carried some virulence genes, especially the alpha-hemolysin (*hla*) gene which was detected at 90.4%. Diverse virulence gene profiles were also reported from different food categories worldwide, such as fishery products in Galicia [29], raw and processed food commodities in Shanghai [31], milk products in South Africa [32] and retail chicken in Egypt [33]. Results of the present study and other published reports suggest that various toxins contribute to the pathogenic potential of *S. aureus* and constitute a risk for consumers’ health.

In this study, sixteen *S. aureus* isolates (30.8%) harboured at least one virulence SE or SEl gene, hence demonstrating the potential toxigenic and pathogenicity of these isolates. SEs classical types (SEA to SEE) were reported in 95% of staphylococcal food poisoning while new identified SEls were responsible for the remaining 5% of food poisoning outbreaks [34]. In addition, the presence of the *egc* cluster (*seg-sei-sem-sen-seo*) was detected in 5.8% of *S. aureus* isolates in this study and this was also observed in food and clinical isolates by other investigators [10,23,24,25,26,27,28,29,30,31,32,33]. The role of the *egc*-encoded superantigens has not been fully elucidated, but a remarkable reduced potency in neutralizing capacity compared to classical SEs was reported by Holtfreter *et al.* [35]. Surprisingly, the study demonstrated that these superantigens were inhibited in less than 10% of human sera, thus suggesting a potential for increased severity of clinical diseases. Recently, food-poisoning outbreaks in Korea and China have been linked to *seg-sei* positive *S. aureus* isolates [36]. Thus, the results from this study in combination with reported publications indicate that the pathogenic role of the *egc* gene cluster should not be underestimated, and further studies should be carried out to elucidate its contribution to pathogenicity of *S. aureus* infections.

Hemolysin genes—*hla* (90.4%), *hld* (59.6%), *hlgv* (30.8%) and leukocidin *lukDE* (38.5%) were the most commonly detected genes in this study, and a high incidence of *hla-hld* combination was confirmed in more than 60% of the strains. These non-enterotoxin *hla, hld* and *lukED* genes were reported to be responsible for staphylococcal food-poisoning outbreaks in China [37]. Hemolysin genes were also observed frequently in food isolates in China [38] and Asturias, Spain [39]. It is noteworthy that isolates recovered from milk of cows with subclinical mastitis in Asturias harboured *hla* (100%), *hlgv* (100%) and *hld* (94.2%) genes [39]. These studies indicate the potential of *S. aureus* in producing cytotoxins (hemolysin and leukotoxin genes) leading to illness in host. *S. aureus* isolates producing the ETA, TSST-1 and lukPV toxins were also detected in this study, which is in agreement with Cha *et al.* [40], who reported 12% and 1.2% prevalence of TSST-1 and ETA in *S. aureus* isolates recovered from stool samples of staphylococcal food-poisoning affected patients in Korea. The presence of these three genes was also reported in RTE sushi, kimbab, California rolls [41] and RTE raw fish [5]. Recently, Song *et al.* [31] showed the presence of lukPV toxins in fresh meats, raw milk, bean products and frozen foods.

Antibiotic-resistant *S. aureus* involved in food contamination has been detected worldwide. Resistance to tetracycline is common among *S. aureus* food isolates but the rates vary greatly [42,43]. In this study, *S. aureus* resistance to tetracycline was at 28.8% with a higher isolation detected in sushi and sashimi samples. A low percentage of multidrug-resistance (MDR) S. *aureus* isolates (3.8%, 2/52) was identified based on the MDR criteria (defined as acquired non-susceptibility to at least one agent in three or more antimicrobial categories) [44]. One isolate presented a MDR profile in of FOX, TE, VA, E, SXT and C. Another isolate showed resistance to the full panel of antibiotics tested—FOX, CN, AK, TE, VA, E, CIP, SXT, C and CFP. Other investigators have also reported the presence of MDR isolates in ready-to-eat raw fish, infant formula milk and cereal [5,45]. Antibiotic resistant *S. aureus* that jeopardizes public health has been reported in numerous foods that can be passed to humans through the food chain [46]. With increase in antibiotic resistance, the use of natural occurring compounds as antimicrobials in the food industry has gained attention. In particular, carvacrol, cinnamon oil, and oregano oil were reported to be active against antibiotic resistant *S. aureus* [47]. However, further evaluation is required before these compounds can be incorporated into food products.

One limitation in this study is that the results only showed the occurrence of *S. aureus* in sushi and sashimi samples, and did not take into account quantitative assessment of this pathogen. Information from the Food and Drug Administration (2011) states that “*S. aureus* toxin does not normally reach levels that will cause food poisoning until the number of pathogens reach 10^4^ to 10^5^ per gram” [48]. Thus, a quantitative contamination level of this pathogen should be carried out in order to provide better microbial risk assessment for public health protection.

## 5. Conclusions

The results from this study show *S. aureus* contamination in 26% (52/200) of the sushi and sashimi samples. A high incidence of virulence genes was identified in 96.2% of the *S. aureus* isolates with confirmation of 20 different virulence gene profiles. The SEs and SEls genes responsible for staphylococcal food poisoning were detected in 30.8% of the *S. aureus*, thus confirming the toxigenic and pathogenic capacity of the *S. aureus* isolates. *S. aureus* producing hemolysins (*hla, hld, hlgv*) as well as ETA, TSST-1, lukPV and lukDE toxins were also detected, and this indicates that *S. aureus* contamination can result in other disorders besides food poisoning. Tetracycline resistance was commonly observed at 28.8%, resistance to at least one antibiotic tested was detected at 34.6% while multiple drug resistance was much lower at 3.8%. There is currently no published data on *S. aureus* contamination and its virulence gene profile in food items in Malaysia. Raw seafood used in sushi and sashimi preparation in Malaysia is generally harvested from sea locations. There are no directives for seafood collection and different outlets possess individual seafood sources. In addition, guidelines for food preparation vary with different outlets as there are no standard guidelines in Malaysia yet. Thus, in conclusion the results of this study highlight the necessity for more detailed investigations for improved consumer protection and public health safety.

## Supplementary Materials

The following are available online at www.mdpi.com/1660-4601/13/2/199/s1, Table S1. Primer nucleotide sequences for DNA amplification and amplified amplicon sizes of 32 virulence genes of *Staphylococcus aureus.*
