# Supplementary material for: Virulence Factors and Antibiotic Susceptibility of Staphylococcus aureus Isolates in Ready-to-Eat Foods: Detection of S. aureus Contamination and a High Prevalence of Virulence Genes
Source: Int J Environ Res Public Health. 2016 Feb 5;13(2):199. doi: 10.3390/ijerph13020199 (PMC4772219; doi:10.3390/ijerph13020199)
Supplement: Supplementary file 1 [file ijerph-13-00199-s001.pdf]

Supplementary Materials: Virulence Factors and Antibiotic Susceptibility of *Staphylococcus aureus* Isolates in Ready-to-Eat Foods: Detection of *S. aureus* Contamination and a High Prevalence of Virulence Genes

Suat Moi Puah, Kek Heng Chua and Jin Ai Mary Anne Tan

Table S1. Primer nucleotide sequences for DNA amplification and amplified amplicon sizes of 32 virulence genes of *Staphylococcus aureus*.

Gene	Upstream Primer Sequence (5′-3′)	Downstream Primer Sequence (5′-3′)	Amplicon Size (bp)	Reference
sea	GGTTATCAATGTGCGGGTGG	CGGCACTTTTTTCTCTTCGG	102	[1]
seb	GTATGGTGGTGTAACTGAGC	CCAAATAGTGACGAGTTAGG	164	[1]
sec	AGATGAAGTAGTTGATGTGTATGG	CACACTTTTAGAATCAACCG	451	[1]
sed	CCAATAATAGGAGAAAATAAAAG	ATTGGTATTTTTTTTCGTTC	278	[1]
see	AGGTTTTTTCACAGGTCATCC	CTTTTTTTTCTTCGGTCAATC	209	[1]
seg	CCAGATTCAAATGCAGAACC	TGCTATCGACACACTACAACC	704	[2]
seh	CGAAAGCAGAAGATTTACACG	GACCTTTACTTATTTCGCTGTC	495	[2]
sei	GACAACAAAACTGTCGAAACTG	CCATATTCTTTGCCTTTACCAG	630	[2]
sej	CATCAGAACTGTTGTTCCGCTAG	CTGAATTTTACCATCAAAGGTAC	142	[3]
sek	CGCTCAAGGCGATATAGGAA	GGTAACCCATCATCTCCTGTGT	570	[4]
sel	CACCAGAATCAC ACCGCT TA	CTGTTTGATGCTTGCCATTG	240	[5]
sem	CTATTAATCTTTGGGTTAATGGAGAAC	TTCAGTTTCGACAGTTTTGTTGTCAT	326	[6]
sen	ATGAGATTGTTCTACATAGCTGCAAT	AACTCTGCTCCCACTGAAC	680	[6]
seo	AGTTTGTGTAAGAAGTCAAGTGTAGA	ATCTTTAAATTCAGCAGATATTCCATCTAAC	180	[6]
sep	GAATTGCAGGGAACTGCTTT	ACCAACCGAATCACCAGAAG	537	[4]
seq	GAACCTGAAAAGCTTCAAGGA	CCAGTTCCGGTGTAAAACAAA	509	[4]
ser	TTCAGTAAGTGCTAAACCAGATCC	CTGTGGAGTGCATTGTAACGCC	367	[7]
ses	TTCAGAAATAGCCAATCATTTCAA	CCTTTTTGTTGAGAGCCGTC	195	[8]
set	GGTGATTATGTAGATGCTTGGG	TCGGGTGTTACTTCTGTTTGC	170	[8]
seu	ATGGCTCTAAAATTGATGGTTCTA	GCCAGACTCATAAGGCGAACTA	409	[7]

Table S1. *Cont.*

Gene	Upstream Primer Sequence (5′-3′)	Downstream Primer Sequence (5′-3′)	Amplicon Size (bp)	Reference
eta	ACTGTAGGAGCTAGTGCATTTGT	TGGATACTTTTGTCTATCTTTTTCATCAAC	190	[6]
etb	ATATCAACGTGAGGGCTCTAGTAC	CAGATAAAGAGCTTTATACACACATTAC	612	[6]
etd	CGCAAATACATATGAAGAATCTGA	TGTCACCTTGTTGCAAATCTATAG	452	[9]
tst	ACCCCTGTTCCCTTATCATC	TTTTCAGTATTTGTAACGCC	326	[1]
lukM	TGGATGTTACCTATGCAACCTAC	GTTCGTTTCCATATAATGAATCACTAC	780	[6]
lukED	TGAAAAAGGTTCAAAGTTGATACGAG	TGTATTCGATAGCAAAAGCAGTGCA	269	[6]
lukPV	ATCATTAGGTAAAATGTCTGGACATGATCCA	GCATCAAGTGTATTGGATAGCAAAAGC	433	[10]
hla	CTGATTACTATCCAAGAAATTCGATTG	CTTTCCAGCCTACTTTTTTATCAGT	209	[6]
hlb	GTGCACTTACTGACAATAGTGC	GTTGATGAGTAGCTACCTTCAGT	309	[6]
hld	AAGAATTTTTATCTTAATTAAGGAAGGAGTG	TTAGTGAATTTGTTCACTGTGTCGA	111	[6]
hlg	GTCAYAGAGTCCATAATGCATTTAA	CACCAAATGTATAGCCTAAAGTG	535	[6]
hlg-2	GACATAGAGTCCATAATGCATTYGT	ATAGTCATTAGGATTAGGTTTCACAAAG	390	[6]

References

1. Mehrotra, M.; Wang, G.; Johnson, W.M. Multiplex PCR for detection of genes for *Staphylococcus aureus* enterotoxins, exfoliative toxins, toxic shock syndrome toxin 1, and methicillin resistance. *J. Clin. Microbiol*. **2000**, *38*, 1032–1035.
2. Mclauchlin, J.; Narayanan, G.L.; Mithani, V.; O'neill, G. The detection of enterotoxins and toxic shock syndrome toxin genes in *Staphylococcus aureus* by polymerase chain reaction. *J. Food Prot*. **2000**, *63*, 479–488.
3. Monday, S.R.; Bohach, G.A. Use of multiplex PCR to detect classical and newly described pyrogenic toxin genes in staphylococcal isolates. *J. Clin. Microbiol*. **1999**, *37*, 3411–3414.
4. Srinivasan, V.; Sawant, A.A.; Gillespie, B.E.; Headrick, S.J.; Ceasaris, L.; Oliver, S.P. Prevalence of enterotoxin and toxic shock syndrome toxin genes in *Staphylococcus aureus* isolated from milk of cows with mastitis. *Foodborne Pathog. Dis*. **2006**, *3*, 274–283.
5. Cremonesi, P.; Luzzana, M.; Brasca, M.; Morandi, S.; Lodi, R.; Vimercati, C.; Agnellini, D.; Caramenti, G.; Moroni, P.; Castiglioni, B. Development of a multiplex PCR assay for the identification of *Staphylococcus aureus* enterotoxigenic strains isolated from milk and dairy products. *Mol. Cell. Probes* **2005**, *19*, 299–305.
6. Jarraud, S.; Mougel, C.; Thioulouse, J.; Lina, G.; Meugnier, H.; Forey, F.; Nesme, X.; Etienne, J.; Vandenesch, F. Relationships between *Staphylococcus aureus* genetic background, virulence factors, agr groups (alleles), and human disease. *Infect. Immun*. **2002**, *70*, 631–641.
7. Hwang, S.Y.; Kim, S.H.; Jang, E.J.; Kwon, N.H.; Park, Y.K.; Koo, H.C.; Jung, W.K.; Kim, J.M.; Park, Y.H. Novel multiplex PCR for the detection of the *Staphylococcus aureus* superantigen and its application to raw meat isolates in Korea. *Int. J. Food Microbiol*. **2007**, *117*, 99–105.
8. Ono, H.K.; Omoe, K.; Imanishi, K.; Iwakabe, Y.; Hu, D.L.; Kato, H.; Saito, N.; Nakane, A.; Uchiyama, T.; Shinagawa, K. Identification and characterization of two novel staphylococcal enterotoxins, types S and T. *Infect. Immun*. **2008**, *76*, 4999–5005.
9. Nakaminami, H.; Noguchi, N.; Ikeda, M.; Hasui, M.; Sato, M.; Yamamoto, S.; Yoshida, T.; Asano, T.; Senoue, M.; Sasatsu, M. Molecular epidemiology and antimicrobial susceptibilities of 273 exfoliative toxin-encoding-gene-positive *Staphylococcus aureus* isolates from patients with impetigo in Japan. *J. Med. Microbiol*. **2008**, *57*, 1251–1258.
10. Lina, G.; Piemont, Y.; Godail-Gamot, F.; Bes, M.; Peter, M.O.; Gauduchon, V.; Vandenesch, F.; Etienne, J. Involvement of Panton-Valentine leukocidin-producing *Staphylococcus aureus* in primary skin infections and pneumonia. *Clin. Infect. Dis.* **1999**, *29*, 1128–1132.

© 2016 by the authors; licensee MDPI, Basel, Switzerland. This article is an open access article distributed under the terms and conditions of the Creative Commons by Attribution (CC-BY) license (http://creativecommons.org/licenses/by/4.0/).
